# Can household energy efficiency dampen crude oil price volatility in the United States?

**DOI:** 10.1371/journal.pone.0307840

**Published:** 2025-01-21

**Authors:** Ojonugwa Usman, Oktay Ozkan, Chinazaekpere Nwani, Festus Victor Bekun, Andrew Adewale Alola

**Affiliations:** 1 Department of Economics, Istanbul Ticaret University, Istanbul, Turkey; 2 University of Economics and Human Sciences in Warsaw, Warszawa, Poland; 3 Adnan Kassar School of Business, Lebanese American University, Beirut, Lebanon; 4 Department of Business Administration, Faculty of Economics and Administrative Sciences, Tokat Gaziosmanpasa University, Tokat, Turkey; 5 Department of Economics and Development Studies, Alex Ekwueme Federal University, Ndufu-Alike, Ebonyi State, Nigeria; 6 Department of Logistics Management, Faculty of Economics Administrative and Social Sciences, Istanbul Gelisim University, Istanbul, Turkey; 7 Department of Economics, Adnan Kassar School of Business, Lebanese American University, Beirut, Lebanon; 8 Faculty of Economics and Administrative Sciences, Recep Tayyip Erdogan University, Rize, Türkiye; 9 Research Center of Development, Economics, University of Economics (UNEC), Baku, Azerbaijan State, Azerbaijan; 10 University of Inland Norway, Elverum, Norway; 11 Faculty of Economics, Administrative and Social Sciences, Nisantasi University, Istanbul, Turkey; University of Balamand, LEBANON

## Abstract

Even though the effect of oil price shocks on macroeconomics has been extensively investigated, the literature on how efficiency in household energy use affect crude oil price volatility is yet explored. This study unveils whether household energy efficiency lower crude oil price volatility asymmetrically in the United States using the historical and forecast dataset that spans from 1970:Q1-2040:Q1. Applying the multivariate case of Quantile-on-Quantile Regression, the empirical results show that household energy efficiency dampens crude oil price volatility with a stronger connection in quantiles before the median quantiles of crude oil price volatility. However, the effect of household energy efficiency decreases with an increase across quantiles of the crude oil price volatility. The results further show that energy-related CO_2_ emissions and retail electricity price intensify crude oil price volatility with varying effects across quantiles. These findings are similar to the sensitivity analysis and robustness checks. Overall, the policy implication of our findings is that government and policymakers need to demonstrate unequivocal commitments to improving not only energy-efficient practices at household level but also to mitigate energy-related environmental disasters.

## 1. Introduction

In the face of the challenges of energy security and environmental sustainability, comprehending the intricate relationship between energy consumption patterns and the dynamics of the global oil market has grown increasingly crucial. Crude oil prices are known for their unpredictability, often experiencing sharp fluctuations in response to various global events and economic factors. Extensive investigations have been undertaken to identify the determinants of crude oil price volatility [1], with research focusing on areas such as geopolitical tensions, supply disruptions, changes in global demand, and macroeconomic conditions [1, 2]. Natural disasters, geopolitical conflicts, and shifts in oil production and exploration can lead to significant price swings, affecting energy markets worldwide. Therefore, understanding the factors contributing to crude oil price volatility has been a major focus of research due to its far-reaching implications for energy security, inflation rates, consumer spending, and overall economic stability [1, 3, 4].

This study aims to explore the intricate dynamics between household energy efficiency and crude oil price volatility, investigating how efficiency in household-level energy use may impact oil price movements. To conduct a policy-informed analysis, this research relies on household energy efficiency trends in the United States of America (hereafter referred to as the United States). As one of the largest global energy consumers, the United States relies significantly on crude oil to meet its energy needs. Consequently, the international oil market may be susceptible to energy demand shocks originating from the United states. The urgent need to address climate change and reduce greenhouse gas emissions has prompted the United States to explore strategies for enhancing energy efficiency across various sectors. Improving household energy efficiency has emerged as a crucial aspect of this broader goal, given that residential energy consumption constitutes a substantial portion of the economy’s overall energy demand [5]. The strategies implemented thus far appear to yield positive results, with average energy use per household declining by about 10% since 2005 [5]. This decline suggests an improvement in households’ capacity to optimize their energy consumption patterns, achieving the same comfort and functionality with reduced energy usage. Various means contribute to this improvement, including the use of energy-efficient appliances, home insulation measures, adoption of renewable energy sources, and adjustments in individual behavioural patterns. Cultivating a culture of energy efficiency at the household level has the potential to lead to reduced energy consumption and, consequently, contribute to broader sustainability goals [4].

The impact of improvement in household energy use on crude oil price volatility is a topic that carries significant implications for energy policy, economic stability, and environmental sustainability. The intricate relationship between household energy-related efficiency and crude oil price volatility stems from a complex interplay of factors. Firstly, improving energy efficiency at the household level has the potential to reduce overall energy demand, thereby potentially decreasing the country’s reliance on imported crude oil [6]. Reduced demand for oil, in turn, could exert downward pressure on global crude oil prices. Lower demand from a major consumer can contribute to a decrease in the international benchmark prices. Secondly, if energy-efficiency measures in the United States households lead to a shift away from traditional fossil fuels like crude oil towards cleaner alternatives such as natural gas, renewables, or electric vehicles, this could have a two-fold impact. First, it could decrease demand for crude oil in the United States, putting downward pressure on global prices. Second, it might increase demand for other energy sources, potentially impacting the prices of those commodities. Thirdly, energy markets are influenced not only by supply and demand but also by market perception and investor sentiment. If the implementation of measures for the U.S. energy-efficiency is seen as a broader trend toward reducing dependence on crude oil, it could affect market sentiment and lead to increased volatility in crude oil prices as traders and investors react to changing market dynamics.

As the United States strives to achieve a low-carbon and sustainable energy future, the efficacy of household/residential energy-related efficiency in dampening crude oil price volatility emerges as a key focus of research and policy discussions. This study aims to use both historical and forecast data to provide valuable insights for developing energy policies that promote price stability in the oil market and reduce the nation’s vulnerability to external shocks. The impact of household energy efficiency measures on global crude oil price volatility is still an emerging issue in the dynamics of the international oil market, presenting ample opportunities for this study to make significant headway contributions in the literature. Firstly, this study can enhance our understanding of market behaviour and price movements, extending insights into the drivers behind crude oil price fluctuations and the potential role of efficient energy usage as a mitigating factor. By developing models to forecast the potential impact of several measures of efficient use of energy on future crude oil prices, researchers can offer valuable tools for energy analysts, and investors to anticipate market developments. Secondly, through empirical exploration, researchers can gain valuable insights into the relationship between energy efficiency and energy demand. This understanding can assist policymakers and energy experts in making informed decisions about energy policies, resource allocation, and market dynamics. Additionally, the study can shed light on the broader economic implications of energy efficiency, not only for households but also for the overall economy and financial markets. Understanding how changes in energy demand affect global crude oil prices can provide valuable guidance to investors, businesses, and governments in making better-informed economic decisions.

Our findings demonstrate that crude oil price volatility is affected negatively to efficient use of household energy and positively to changes in energy-related CO_2_ emissions––an indicator of the environment and retail electricity prices––an indicator of substitute energy price in the United States. Furthermore, the effects of household/residential efficient use of energy, energy-related CO_2_ emissions, and prices for retail electricity have asymmetric effects.

The remaining part of the study follows the organization below: Section II which follows the introduction provides a review of relevant literature. Section III describes the econometric model adopted. Section IV presents the data and discusses the empirical results while Section V concludes the paper with policy recommendations. It also suggests future research based on the limitations identified in our study.

## 2. Review of relevant literature and hypothesis development

Numerous researchers have conducted studies to uncover the factors influencing crude oil price volatility. Previous investigations have primarily focused on supply and demand shocks as the key contributors to long-term fluctuations in oil prices [2, 7]. In their study, [7] delve into the impact of oil price volatility on international macroeconomic indicators using a vector autoregression (VAR) system. The research reveals that economic recovery and investment have been significantly influenced by oil price volatility from 2000Q1 to 2020Q4. Notably, business investment and oil prices exhibited resilience during the international economic meltdown, including the recent COVID-19 outbreak. The study emphasizes a robust connection between oil prices and economic growth during global financial crises, with the COVID-19 epidemic leaving the most significant impact. This indicates faster information propagation between the oil market and financial initiatives compared to previous crises. [2] undertakes a comprehensive exploration of factors affecting crude oil price volatility, encompassing commodity attributes, macroeconomics, geopolitics, and alternative energy. Employing a GARCH-MIDAS model with single and multi-factor variants, the research demonstrates that multi-factor models outperform single-factor ones in predictions. The study reveals that supply, demand, inventories, US dollar exchange rate, and geopolitical risk play major roles as long-term influences, while alternative energy has a minor impact.

In a recent study by [8], the connectedness of oil price volatility and the general aspect of energy efficiency was examined by using the case of China and with emphasis on the influence of the coronavirus pandemic. The investigation implemented continuous Generalized Autoregressive Conditional Heteroskedasticity (co-GARCH) and Asymmetric Dynamic Conditional Correlations (ADCC). The investigation found that green fiscal policies influence the relationship between energy efficiency and volatility of the energy mix especially during the pandemic period 2019–2020. Importantly, with the influence of green fiscal policies, the result shows that energy efficiency is connected with (i) oil price volatility with a significance value of 16.4%, (ii) gasoline price volatility with a significance value of 28.7%, (iii) heating oil volatility with a significance value of 18.2% (iii) natural oil price volatility with a significance value of 9.7%, and (iv) natural oil price volatility with a significance value of 34.1%. Similarly, the relationship between energy efficiency in Malaysia and the volatility of oil prices vis-a-vis global oil price uncertainty was examined in the work of [9]. By employing the integration of stochastic approach and computable general equilibrium (CGE) over the period 1980–2017, the findings show that global oil uncertainty affects energy efficiency in Malaysia by changing energy efficiency level between –0.08% and + 0.06% and also changing household welfare in Malaysia between –4.81% and +2.92%.

Moreover, [10] explored the drivers of natural resources commodity price volatility from the perspective of economic and non-economic factors while volatility spillovers between non-conventional energy sources and crude oil were considered by [11]. Importantly, [10] implemented the autoregressive distributed lag (ARDL) alongside other econometric tools and the prices of natural resources commodities for China over the period of 1986 to 2019. The finding reveals that macroeconomic indicators i.e., inflation and GDP growth, and the non-economic indicators such as human capital and social contribution are all positively associated with natural resources commodity prices in China. Similarly, while implementing the spillover examination technique documented by Diebold and [12, 13], the recent study of [11] also examined the aspects of new energy and crude oil contagion in China. Importantly, the finding reveals that net volatility is largely transmitted by new energy resources and largely received by crude oil commodity price, thus justifying the direction of impact from new energy to crude oil price volatility.

However, beyond the above-highlighted studies, the link between crude oil volatility and the specificity of energy use, say household energy efficiency is rarely studied in the literature. Although, while positing the case of the European Union (EU), [14] considered energy efficiency indicators from the economy and sector-wide levels, but failed to specifically provide information about the nature of the relationship between crude oil volatility and household energy use. Clearly, this existing gap in the literature has been covered in the current investigation.

Consider the empirical studies we have reviewed in this study, we formulate the following hypotheses to guide the study:

1. **H**_**1**_**(a):** Improvement in household energy efficiency is associated with a decline in crude oil price volatility
**H**_**1**_**(b):** The effect of household energy efficiency is asymmetric across the conditional quantiles of crude oil price volatility and household energy efficiency
2. **H**_**1**_**(a):** Improvement in energy-related CO_2_ emissions is associated with a decline in crude oil price volatility
**H**_**1**_**(b):** The effect of energy-related CO_2_ emissions is asymmetric across the conditional quantiles of crude oil price volatility and energy-related CO_2_ emissions
3. **H**_**1**_**(a):** Increase in retail electricity prices is associated with a decline in crude oil price volatility
**H**_**1**_**(b):** The effect of retail electricity prices is asymmetric across the conditional quantiles of crude oil price volatility and retail electricity prices

## 3. Nonparametric MQQR model

This section presents a novel methodology based on the Quantile-on-Quantile Regression (QQR) of [15]. The standard QQR model was developed by [15] to uncover the impact of one variable’s quantiles on the dependent variable’s quantiles. The QQR is based on the bivariate model, leaving out the effect of other variables, which could influence changes in the dependent variable. This becomes a serious criticism of QQR in the extant literature [16–19]. In this section, we apply a model developed by [4] which extends the standard QQR model to the multivariate case and refers to it as the Nonparametric Multivariate-QQR model. The Multivariate-QQR model incorporates other independent variables based on theoretical and empirical studies to determine changes in the dependent variable. The Multivariate-QQR technique minimizes the omitted variable bias exposed by the standard QQR. In principle, before we specify the Multivariate-QQR model, let us consider the functional form of the empirical model for this study:

$$LNCOPV=f(LNHEE,{LNCO}_{2},LNREP)$$ (1)

where *LNCOPV* is a dependent variable, and it is apparently expressed in its natural logarithm. The household/residential energy efficiency is perhaps the focal explanatory variable which is also expressed in its natural logarithm (*LNHEE*). Other independent variables include the natural logarithm of energy-related CO_2_ emissions which is a measurement of environmental indicator and *LNREP* as substitute energy prices. The above economic model i.e., Eq (1) is consistent with the previous documentation on the drivers of commodity price and its volatility [20, 21]. Given that prices of commodities (as assets) "jump" to equilibrate goods and money markets [22], oil price volatility is found to be mainly associated with economic agents’ decision-making behaviours to minimize the effect of shocks [23, 24], we reasoned that household behaviours toward the adoption of energy use through energy savings and energy efficiency measures are indeed crucial to oil price volatility. Consequently, the previous Eq (1) is transformed into an econometric model as follows:

$$ln{COPV}_{t}=\alpha_{0}+\beta_{1}ln{HEE}_{t}+\beta_{2}ln{CO}_{2t}+\beta_{3}ln{REP}_{t}+\mu_{t}$$ (2)

where *α*_0_ is the intercept, *t* denotes the time-trend while *μ* signifies the unobserved factors also known as the residual term. The residual term possesses a zero mean with variance *σ*^2^, *ε*_*t*_/*u*_*t*_~*iid*(0,σ^2^). *β*_1_, *β*_2_, and *β*_3_ are scope coefficients of independence variables as stated.

As we have mentioned above, the multivariate QQR is an extension of the QQR model proposed by [15] to the case of multivariate model. Therefore, to accomplish this objective, we assume that *x*_1_, *x*_2_…*x*_*n*_ are predictors, and their effects do not take predetermined forms. The Multivariate QQR model employed in this study can be specified as follows:

$${LNCOPV}_{t}=\alpha_{0}(\theta ,\tau_{1},\tau_{2},\tau_{3})+\beta_{1}(\theta ,\tau_{1})({LNHEE}_{t}-\tau_{1})+\beta_{2}(\theta ,\tau_{2})({LNCO}_{2t}-\tau_{2})+\beta_{3}(\theta ,\tau_{3})({LNREP}_{t}-\tau_{3})+\mu_{t}^{\theta ,\tau_{1},\tau_{2},\tau_{3}}$$ (3)

where *τ*_1_, *τ*_2_, and *τ*_3_ are quantiles of household energy use, energy-related CO_2_ emissions, and retail electricity prices while *θ* simply shows the *θ*-th quantile of the crude oil price volatility. Note that *α*_0_, *β*_1,2,3_, and *μ* are the intercept, slope, and error parameters, respectively, that vary with the quantiles of the dependent and independent variables. It can be seen here that the slope parameters *β*_1_, *β*_2_, *and β*_3_ capture the varying effects of LNHEE, LNCO_2_, and LNREP on LNCOPV at different quantile pairs, and therefore the MQQR is able to unveil potentially nonlinear relationships between LNCOPV and LNHEE, LNCO_2_, and LNREP that cannot be detected by the parametric approach. The MQQR has a nonparametric characteristic as its slope coefficients are not fixed but change with the quantiles of the variables. Furthermore, to select the optimal bandwidth size, we follow [15] by selecting an optimal bandwidth size of 0.05 for the estimations.

**Fig 1:**
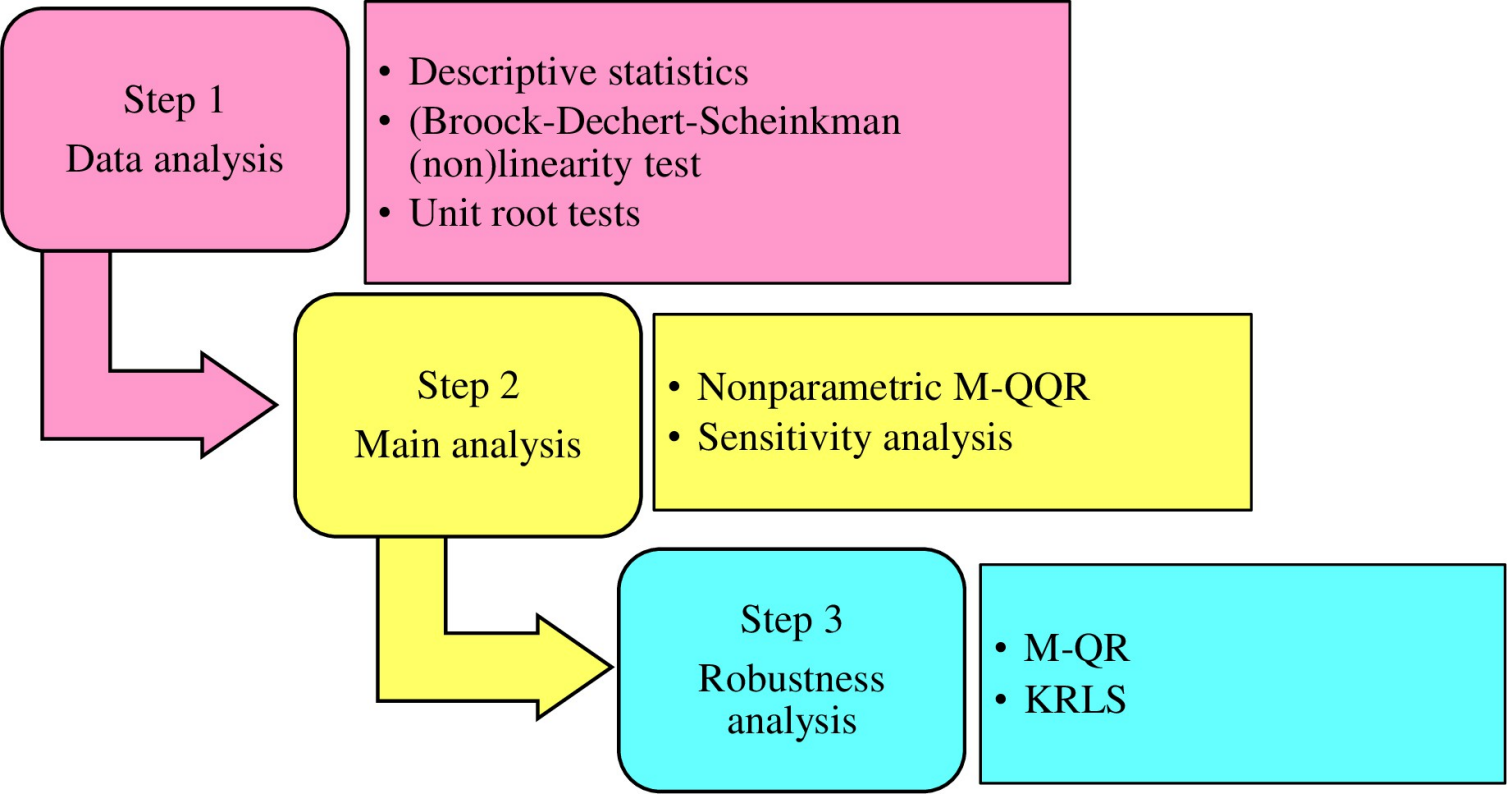
Description of the empirical procedure. By using arrow and squares, the image illustrates the analysis pathway starting from the descriptive statistics and other preliminary tests to the main estimation and robustness test.

Furthermore, since the time series data employed in this study have part that is based on the forecast––we conduct a sensitivity analysis following [19] by re-estimating the Multivariate QQR model using only the historical component of the data employed in this study. Overall, the flow chart of the research is presented in Fig 1.

## 4. Data description and empirical results

### 4.1. Data description

In this section, we demonstrate empirically how crude oil price volatility responds to household energy-related efficiency in the United States over the various quantiles, applying data, which span the period 1970Q1 to 2040Q4. The annual frequency variables were collected and then converted to quarterly frequency using the quadratic match sum. This method of conversion is the most preferred method of converting low frequency data to high frequency data as provided by [4]. Specifically, the data for this study have two parts, namely; the historical part which covers the period 1970 to 2020, and the forecast part which covers from 2021 to 2040. The dependent variable is the crude oil price volatility (LNCOPV) while the core independent variable is the household energy efficiency (LNHEE). Other control variables include energy-related CO_2_ emissions (LNCO_2_), an environmental indicator, and retail electricity prices (LNREP), which capture the substitute energy prices. The data for this study are all sourced from the Global Energy Institute (GEI) database and transformed into their logarithm forms. Table 1 presents the description of data, measurement, and sources as follows:

**Table 1:** Data description.

Data name	Symbol	Measurement	Source
Crude Oil Price Volatility	COPV	Change in year-to-year price	Global Energy Institute (GEI)
Household/Residential Energy Efficiency	HEE	Million Btu/household	Global Energy Institute (GEI)
Energy-Related CO_2_ Emissions	CO_2_	MMTCO_2_	Global Energy Institute (GEI)
Retail-Electricity Prices	REP	cents/kWh (2018$)	Global Energy Institute (GEI)

**Note:** Authors’ creation

### 4.2 Visual properties of the data

**Fig 2:**
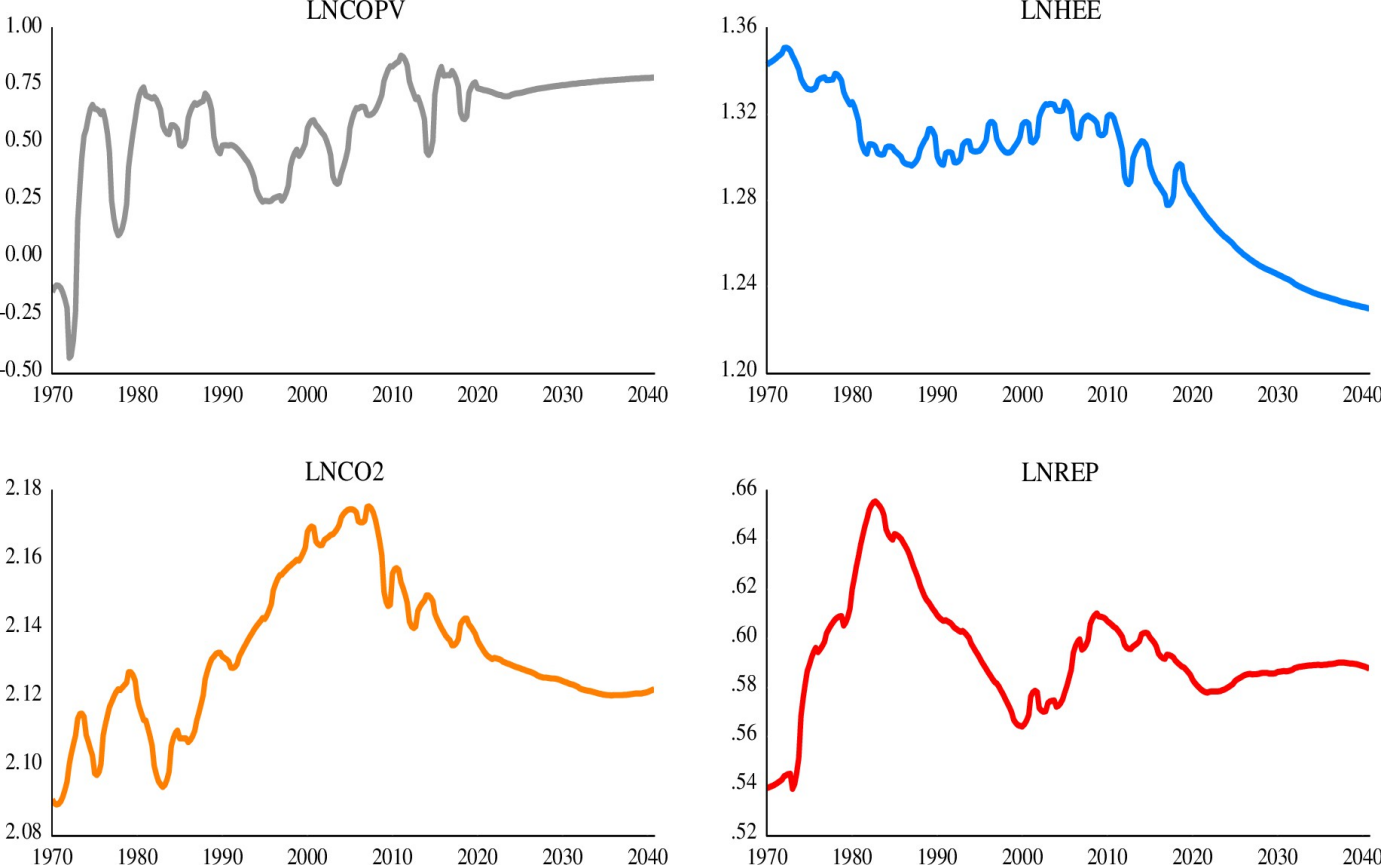
Time series graph of the variables. The vertical and horizontal axes are respectively the values (in logarithm) and years of each series. Upper left panel image is the Crude Oil Price Volatility, Upper right panel is the Household/Residential Energy Efficiency, lower left panel is the Energy-Related CO2 Emissions, and lower right panel is the Retail-Electricity Prices.

Fig 2 presents and describes the evolution of the time series used for this study. As can be seen, all the variables exhibit fluctuations over time. This volatility is more evident in the case of crude oil price volatility. However, the volatility is minimized in the forecast part of the series due to an improved energy efficiency. The trend of household energy efficiency and energy-related CO_2_ emissions show a downward slope after the global financial crisis in 2008/2009. This suggests a decline in the level of energy consumption intensity and environmental degradation. Furthermore, the prices of electricity continued to rise starting from 1973 through the 1980s due to energy shortages arising from the turmoil in the Middle East. This eventually falls continuously until the year 2000, after which the prices of retail electricity become relatively stable.

### 4.3. Descriptive statistics

The descriptive statistics are performed for the logarithmic quarterly series are revealed in Table 2. As we can vividly see, the LNCO_2_ has the largest mean of 2.132 with a minimum score of 2.088 to a maximum score of 2.175. This is followed by the LNHEE with a mean score of 1.293 ranging from 1.229 to 1.351, and the LNREP with a mean score of 0.593 which ranges from a minimum score of 0.538 to a maximum score of 0.655. The lowest mean score is ascribed to LNCOPV which has the mean of 0.579 while the minimum value is -0.436 and the maximum value is 0.881. Furthermore, the values of standard deviation which are close to zero in all the variables, portray that the data points are clustered around the mean. The skewness for LNCOPV and LNHEE is negative, suggesting that these variables are negatively skewed while LNCO_2_ and LNREP are positive, indicating that these variables are positively skewed. Moreover, the value of the skewness of LNCOPV is -1.761, which suggests that the distribution of this variable is highly skewed. For the kurtosis, it is revealed that all the variables exhibit a positive kurtosis with evidence of excess kurtosis in the case of LNCOPV and LNREP. Consequently, the probability values of Jarque-Bera statistics advanced by [25] reject the null hypothesis of normal distribution in all the variables. The non-normality of variables is an indication of nonlinearity, which is a preliminary justification for the use of the quantile-based analysis.

**Table 2:** Descriptive statistics of the logarithmic quarterly series.

	LNCOPV	LNHEE	LNCO2	LNREP
Mean	0.579	1.293	2.132	0.593
Median	0.659	1.302	2.128	0.589
Maximum	0.881	1.351	2.175	0.655
Minimum	-0.436	1.229	2.088	0.538
Std. Dev.	0.242	0.033	0.021	0.023
Skewness	-1.761	-0.476	0.260	0.383
Kurtosis	6.444	2.154	2.496	3.990
Jarque-Bera	287.258***	19.204***	6.209**	18.589***
p-value	0.000	0.000	0.044	0.000

**Note:** *, and ** represent 1% and 5% significance levels

### 4.4. Unit root tests

**Fig 3:**
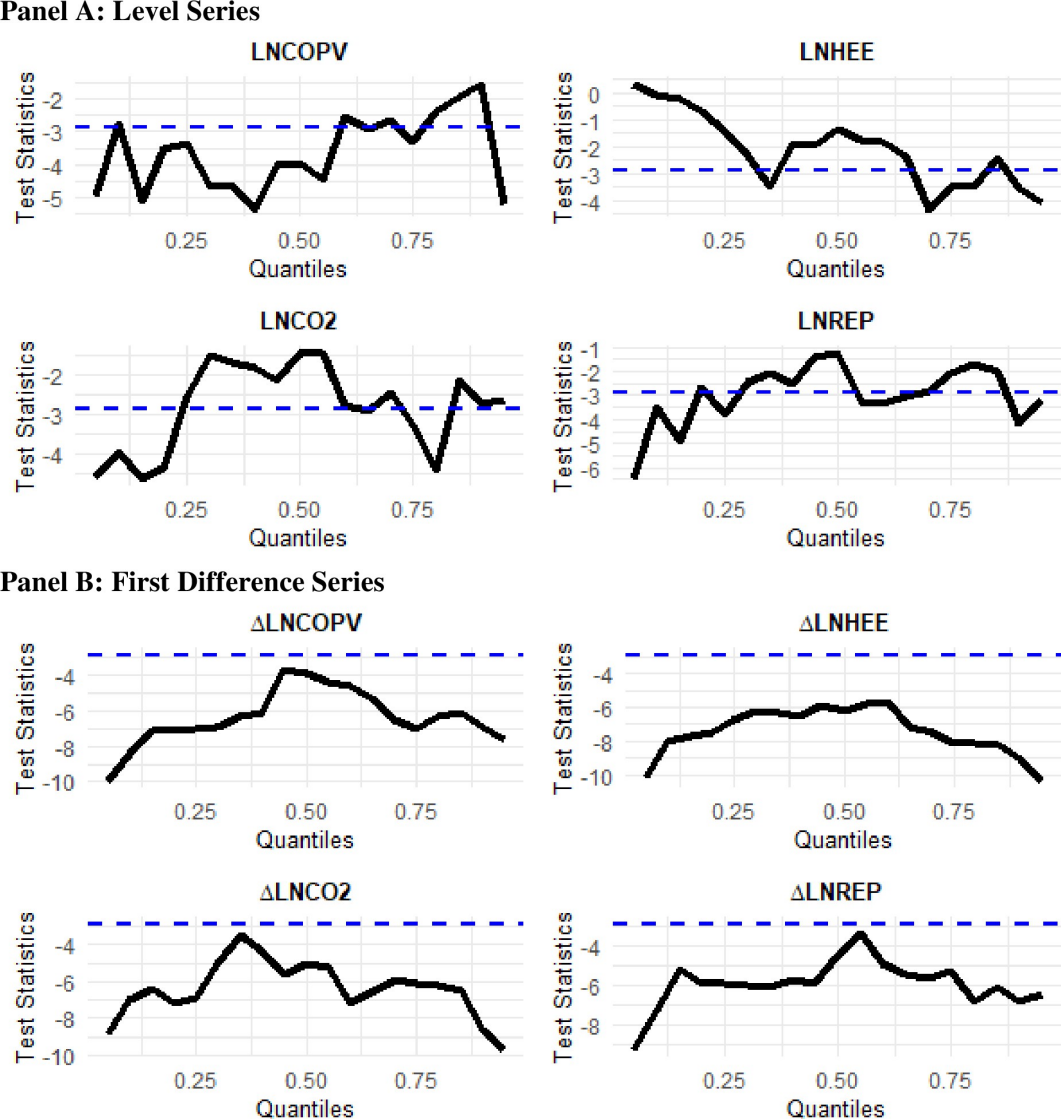
Quantile augmented Dickey-Fuller (QADF) unit root test. The upper and lower panels depict the level and 1^st^ difference unit root tests respectively for the variables Crude Oil Price Volatility, Household/Residential Energy Efficiency, Energy-Related CO_2_ Emissions, and Retail-Electricity Prices.

**Fig 4:**
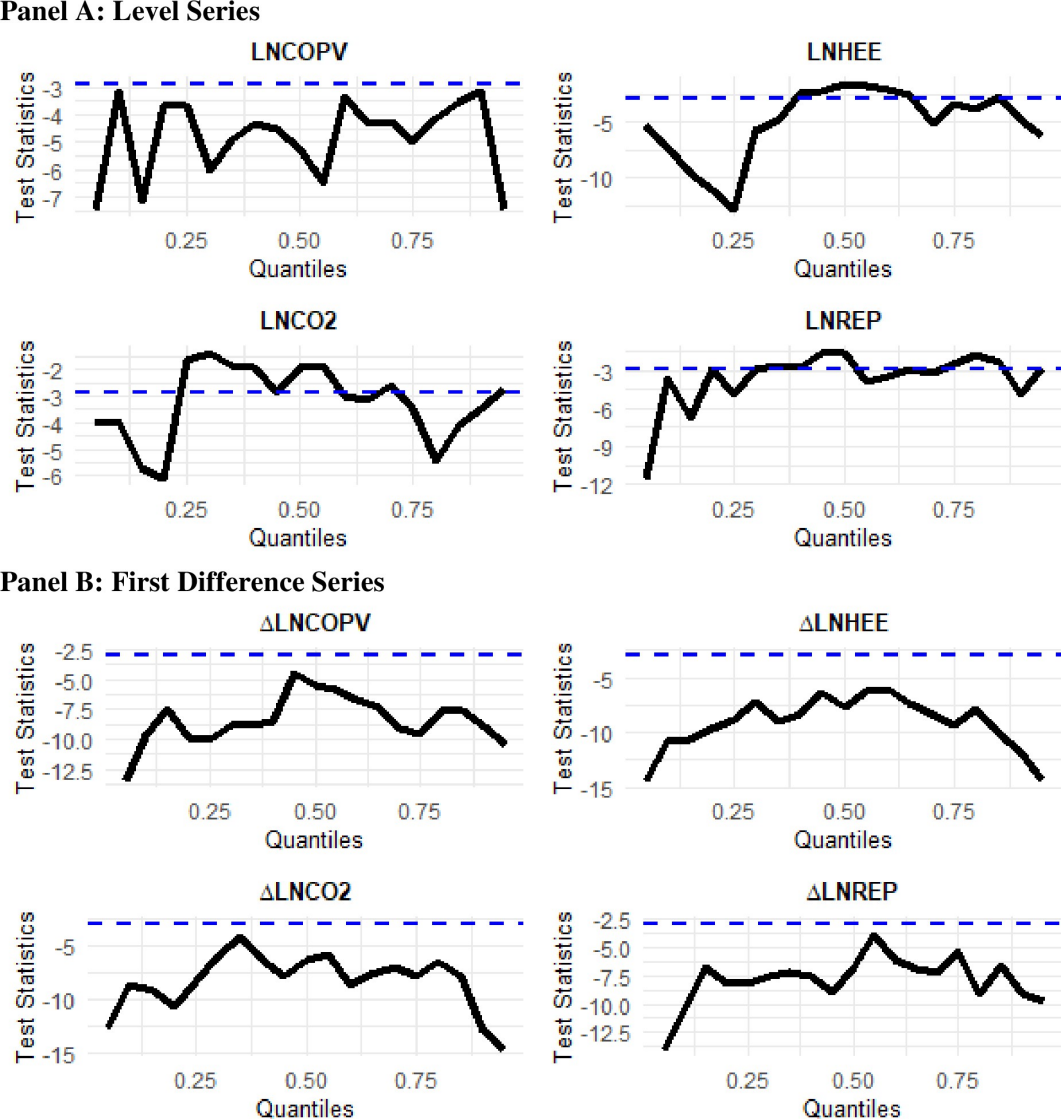
Quantile Phillips-Perron (QPP) unit root test. The upper and lower panels depict the level and 1st difference unit root tests respectively for the variables Crude Oil Price Volatility, Household/Residential Energy Efficiency, Energy-Related CO2 Emissions, and Retail-Electricity Prices.

Since the empirical analysis of the study is based on the quantile-based analysis, we employ the quantile augmented Dickey-Fuller (QADF) and quantile Phillips-Perron (QPP) unit root tests by [26], which is an extension of the traditional ADF and PP unit root tests. These tests detect the possible unit roots at the different quantile levels of the series distribution. The results of the QADF and QPP unit root tests are reported in Figs 3 and 4, respectively. The QDF results displayed in Fig 3 imply that the level series of all variables present a unit root at some of quantiles, whereas their first difference series are stationary at all quantiles. On the other hand, the QPP estimates shown in Fig 3 indicate that only LNCOPV is stationary at level, while other variables are stationary at first difference for all quantile levels. Based on the QADF and QPP unit root tests results, we conclude that all variables are stationary at first difference.

### 4.5. BDS nonlinearity test

Table 3 reports the results of the non(linearity) test based on the BDS advanced by [27]. The null hypothesis/assumption for the test which supports the independent and identical distribution of the residuals could not hold. Therefore, it turns out to be concluded that there is nonlinearity in the relationship that this study seeks to evaluate. This conclusion is also reflected in the descriptive statistics whereof the data distribution is not normal due to high values of skewness and kurtosis with evidence of fat-tailed behaviours. Overall, the evidence of possible asymmetries in the relationship we seek to unveil motivates greatly the use of the Multivariate-QQR approach.

**Table 3:** BDS (Non)linearity test results.

	LNCOPV	LNHEE	LNCO2	LNREP
Dimension 2	31.295***	55.042***	53.466***	29.421***
p-value	0.000	0.000	0.000	0.000
Dimension 3	32.722***	58.429***	56.926***	31.171***
p-value	0.000	0.000	0.000	0.000
Dimension 4	34.522***	62.890***	61.223***	33.333***
p-value	0.000	0.000	0.000	0.000
Dimension 5	37.244***	69.598***	67.437***	36.489***
p-value	0.000	0.000	0.000	0.000
Dimension 6	41.062***	79.011***	76.089***	40.818***
p-value	0.000	0.000	0.000	0.000

**Note:** *** represents 1% significance level.

### 4.6. Multivariate-QQR results

In this sub-section, the results of Multivariate-QQR are indeed divulged and discussed. The analysis here is divided into two parts. The first part is based on the baseline model estimation as already discussed. The baseline estimation of the Multivariate-QQR model is traceable to data spanning from 1970:Q1–2040:Q4 while the second part is what is called sensitivity analysis. This part uses historical data, which only spans from 1970:Q1 to 2020:Q4.

#### 4.6.1. Multivariate-QQR results

**Fig 5:**
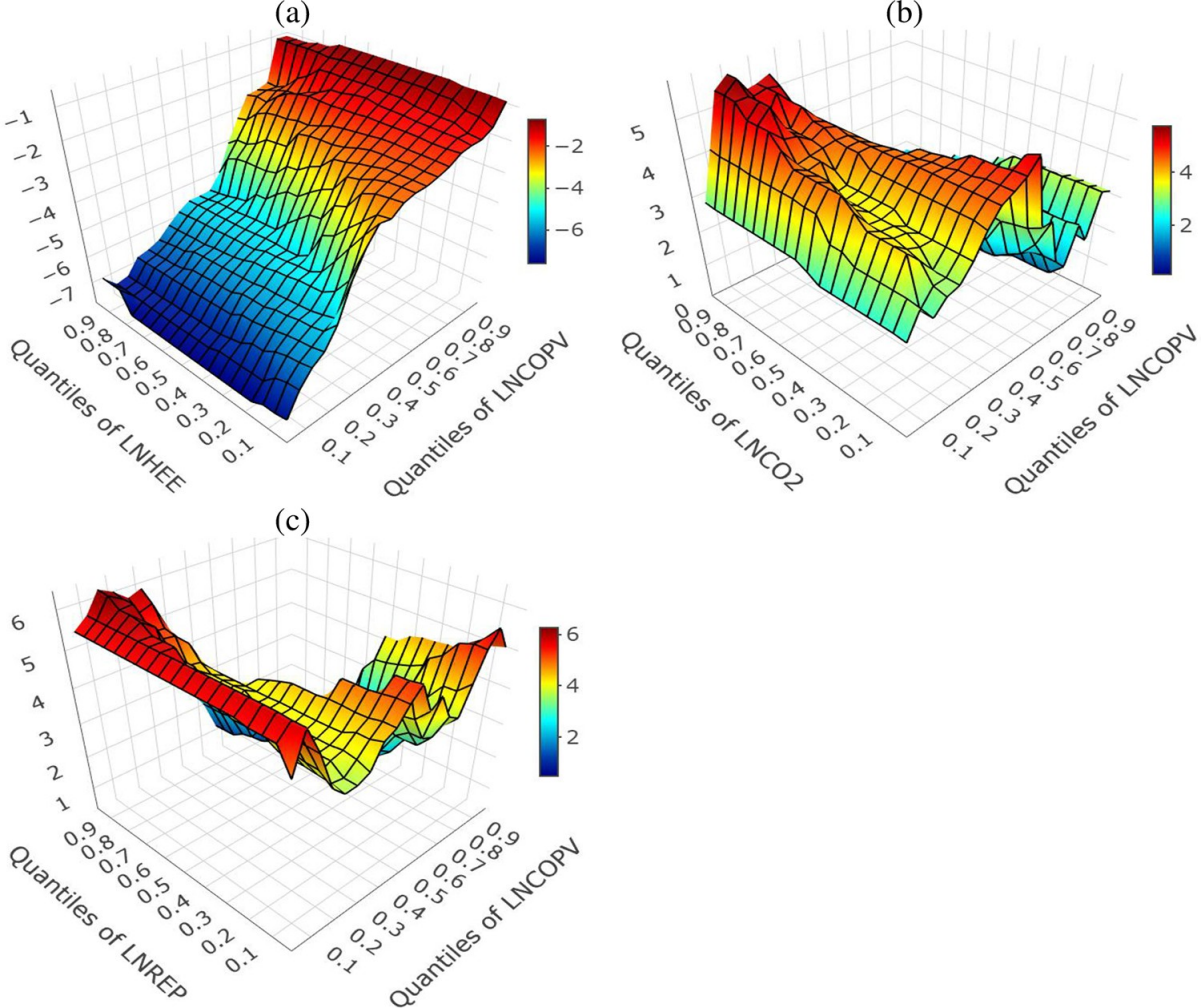
The nonparametric multivariate-QQR result for historical data1970:Q1–2040:Q4. Panel (a) demonstrates the influence of LNHEE on LNCOPV. Panel (b) demonstrate how LNCO2 influences Panel (c) LNCOPV demonstrate the influence of LNREP on LNCOPV.

The nonparametric multivariate-QQR provides results that are reliable for policy analysis and forecast compared to the bivariate QQR because of omitted variable bias [28]. As we can easily see from Fig 5, which shows the slope coefficients estimated based on the Multivariate-QQR method. The results divulge an asymmetric effect of LNHEE, LNCO_2_, and LNREP on LNCOPV. Explicitly, Fig 5(A) demonstrates the influence of LNHEE on LNCOPV. From this figure, it is seen that the influence of LNHEE on LNCOPV is negative across all quantiles with asymmetric effects. Specifically, this negative effect is quite strong in all quantiles of LNHEE (i.e., 0.05–0.95). Particularly, the effect is stronger in quantiles below the median quantiles of the LNCOPV (i.e., 0.05–0.40). Also, we observe that the negative impact of LNHEE on LNCOPV decreases with increasing quantiles of LNCOPV. Further, Fig 5(B) demonstrate how LNCO_2_ influences LNCOPV. This Figure reveals that the effect of theLNCO_2_ on LNCOPV is positive across all quantiles of LNCOPV, although with evidence of asymmetric effects. In specific, a strong positive effect of LNCO_2_ is established in all various quantiles of LNCO_2_ (i.e., 0.05–0.95). Even though the effect is positive, it differs across the quantiles. For instance, a stronger is found in the upper tails of the lower and higher quantiles of LNCOPV and LNCO_2_. Finally, Fig 5(C) divulges the influence of LNREP on LNCOPV. As we can observe, the influence of LNREP on LNCOPV is positive for all quantiles, ranging from 0.05 to 0.95. In specific, the positive influence of LNREP on LNCOPV is stronger in higher quantiles of LNCOPV. In addition, the effects of LNCO_2_ and LNREP are only strong in the higher quantiles.

#### 4.6.2. Sensitivity analysis

**Fig 6:**
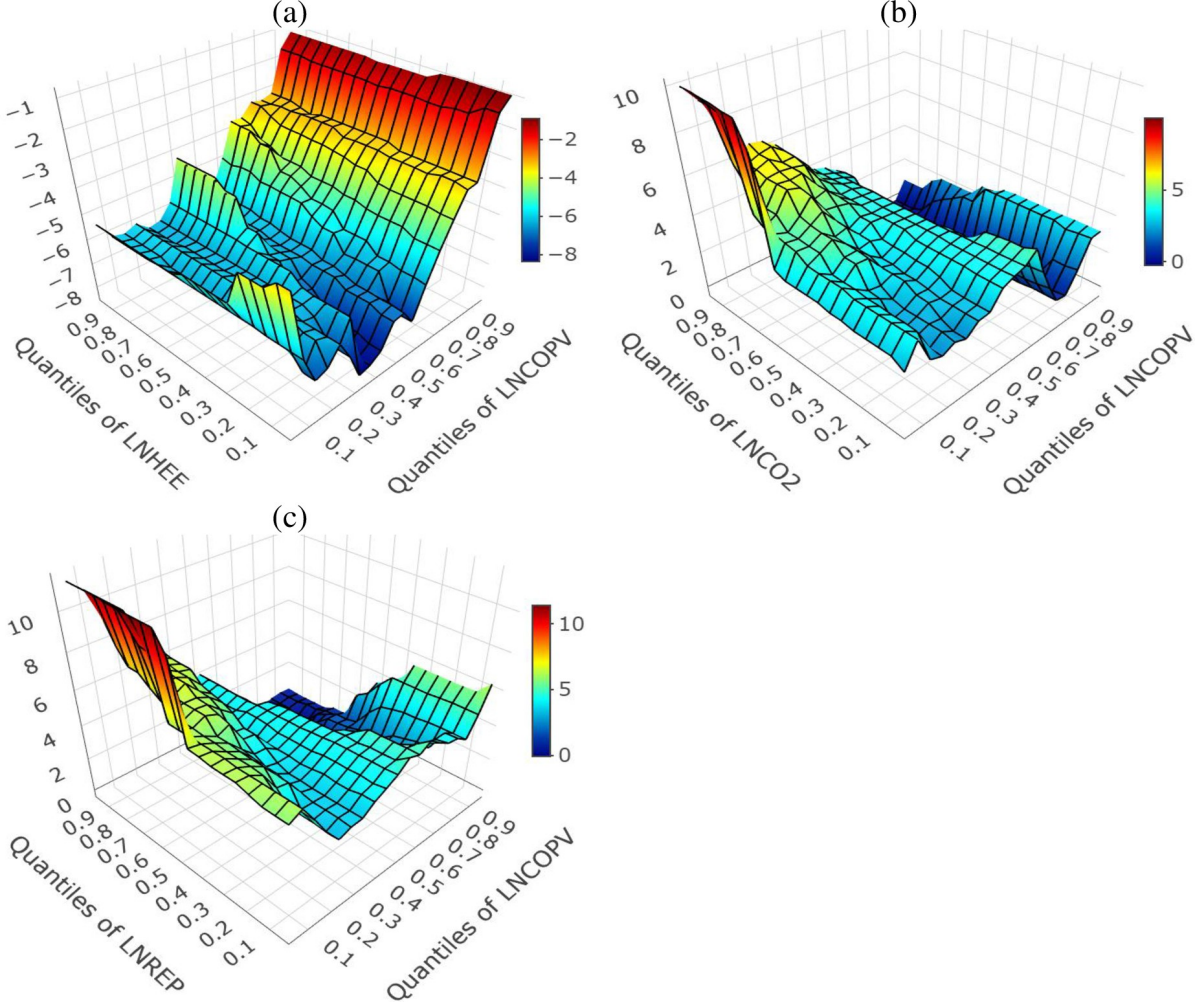
Sensitivity result with nonparametric multivariate-QQR for historical data1970–2040. Panel (a) demonstrates the effect of LNHEE on LNCOPV. Panel (b) demonstrate the effect of LNCO2 on LNCOPV. Panel (c) demonstrate the effect of LNREP on LNCOPV.

Given that the time-series data employed in this study has some forecast component, we check the sensitivity of our estimates based on the historical component of the data i.e. from 1970 to 2020. As indicated by Fig 6(A)-6(C), the LNHEE mitigates LNCOPV in all quantiles i.e. lower, middle, and higher levels of quantiles and the lower quantiles of LNCOPV. On the contrary, the LNCO_2_ and LNREP amplify the LNCOPV in all quantiles with further positive effects at the lower quantiles of LNCOPV. The similarity of the sensitivity results and those obtained based on the baseline model therefore confirm the reliability of the forecast data employed in this study. This also gives credence to policymakers to formulate energy and environmental policies based on the findings and recommendations of this study.

### 4.7. Discussion of empirical findings

The empirical results reveal that LNCOPV responds negatively and asymmetrically to LNHEE in the quantiles of the LNCOPV. Particularly, the effect is stronger in the quantiles slightly below the median quantiles of the LNCOPV (i.e., 0.05–0.40). Also, the negative effect of LNHEE decreases with an increase in LNCOPV across quantiles. The possible reason behind these findings is indeed traceable to improvements in household energy use, particularly, the household appliances and computers, which consequently reduces the amount of energy consumption in the country and dampens the pressure on crude oil production. Lowering energy demand by firms and industries and producing same quantities of commodities (goods and services) implies a lower risk to energy security and improvement in energy efficiency. Specifically, with the recent transition energy policy towards alternative energy, household energy efficiency may play an important role in dampening crude oil price volatility. In the U.S., roughly about 11.5 quadrillion Btu (British thermal units) were consumed only in 2019. This amount is greater than the amount of coal consumption and nuclear energy use for the first one hundred and thirty years ago as demonstrated by the U.S. Energy Information Administration [29]. An increasing pace of energy transition is significantly linked to a downward pressure on energy consumption from fossil fuel sources. Clean and alternative energy, including wind, solar, biomass, hydropower, and geothermal can reduce the average energy use per household and the demand for fossil fuel energy such as crude oil as well as enhance energy-efficient appliances, computers, home insulation measures, adoption of renewable energy sources, and adjustments in individual behavioural patterns. This finding is consistent with [4, 30].

Furthermore, the positive influence of LNCO_2_ on LNCOPV means that an increase in LNCO_2_ would increase not only energy consumption but exert upward pressure on crude oil prices. Consequently, the level of volatility in crude oil prices would increase as well. There are possible reasons for establishing this result. First, the accumulations of CO_2_ emissions and other greenhouse gases are linked to fossil fuel and other traditional energy consumption. Therefore, an increase in LNCO_2_ is a signal of a rise in energy generation, particularly from non-renewable sources such as crude oil, natural gas, coal, etc. This finding is consistent with [31] who established a negative relationship between ecological footprints and electricity consumption in Brazil.

The possible influence of LNREP on LNCOPV is positive. This, of course, suggests that LNCOPV responds to substitute energy prices, i.e. as substitute energy prices such as retail electricity prices increase, crude oil price volatility would also increase in the same direction. This is because a large portion of electricity was generated from fossil fuel sources in the 1970s through 2015, after which emphasis is now placed on clean electricity sources such as nuclear power, hydropower, wind, solar, etc. Therefore, our finding is in agreement with the finding of [31] wherein oil production is closely associated with electricity prices for 15 oil-producing economies. This finding is of course resounding with [32] who show that fossil fuels such as crude oil, coal, and natural gas prices are positively associated with electricity prices in Mexico. On the contrary, our result is inconsistent with [33] who discovered that electricity prices are not significantly related to crude oil prices in the United States.

### 4.8. Robustness checks

**Fig 7:**
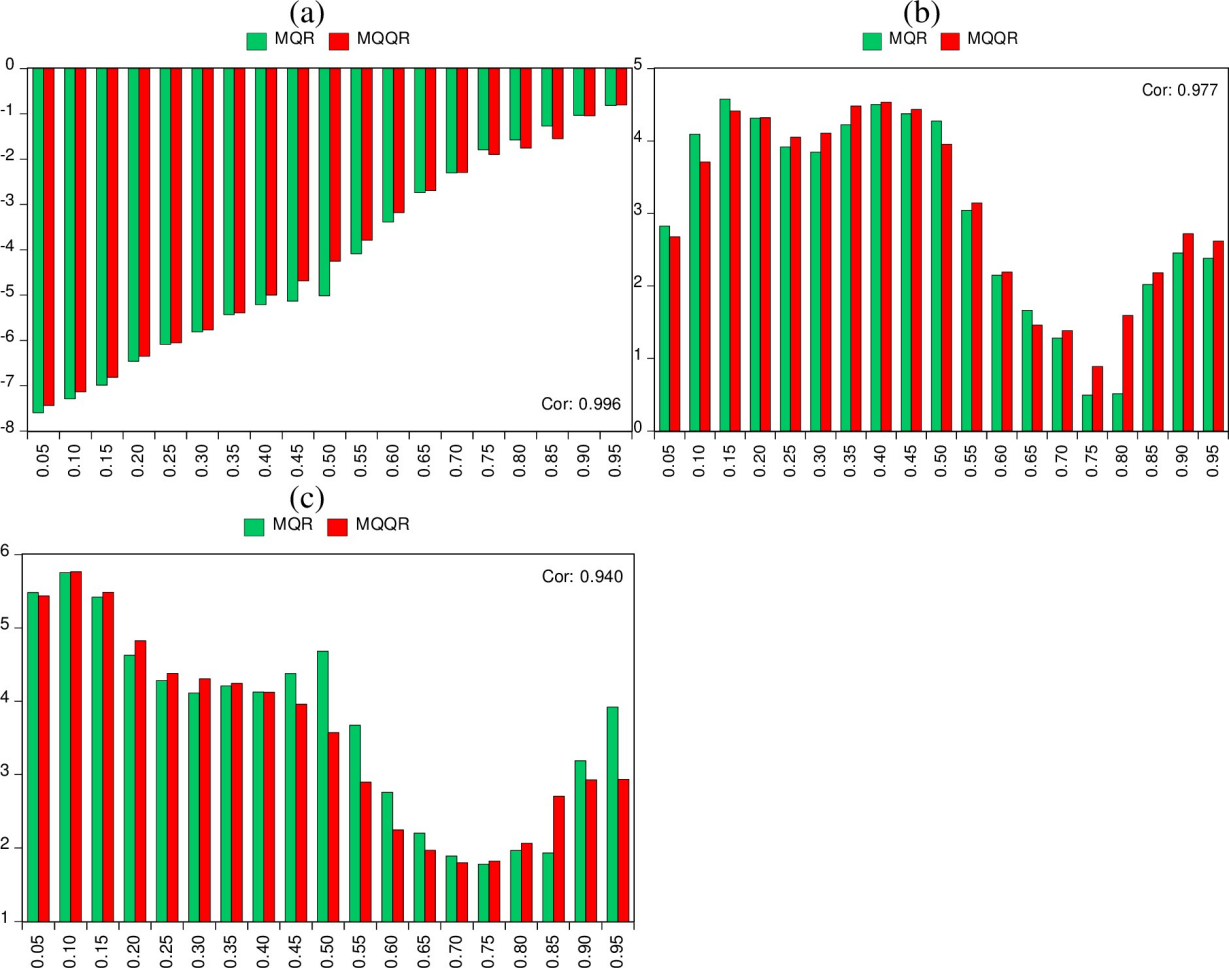
Robustness estimation by comparing multivariate QR and QQR. The midpoint of the Multivariate QR and QQR across the quantiles of crude oil price volatility are employed against LNHEE (in panel a), LNCO2 (in panel a), and LNREP (in panel a). Red and blue colours respectively depict QR and QQR.

For robustness checks, we first compare the estimates of the multivariate-QR model with the average of the Multivariate-QQR estimates. Fig 7(A)-7(C) report the estimates of the Multivariate-QR and perhaps the midpoint of the Multivariate-QQR across the quantiles of crude oil price volatility. Accordingly, the effect of an improvement in household energy utilization on crude oil price volatility is negative, and the trend of the estimates is close to each other. For LNCO_2_ and LNREP, their effects on LNCOPV are not only positive but they exhibit co-movement. These results, therefore, suggest that the results based on the multivariate-QQR are robust.

In furtherance of the robustness checking, we also employ the KRLS machine learning approach by [34]. As divulged in Table 4, the R^2^ estimated is 0.873, which indicates that LNHEE, LNCO_2_, and LNREP explain about 87.3% of the total variations in LNCOPV in the United States. The results confirm that findings based on the baseline and sensitivity models, which clearly show that LNHEE dampens LNCOPV but LNCO_2_ and LNREP promote LNCOPV. Particularly, a 1% increase in LNHEE would result in a 6.593% reduction in the LNCOPV while a 1% increase in LNCO_2_ and LNREP causes LNCOPV to rise by 8.426% and 8.219% respectively if other variables are held constant.

**Table 4:** Average-pointwise marginal effect.

	Average	Standard Error	*t*-value	*p*-value	25%	50%	75%
LNHEE	-6.593***	0.621	-10.616	0.000	-13.073	-3.892	-2.178
LNCO2	8.426***	1.196	7.044	0.000	6.961	10.359	13.592
LNREP	8.219***	0.874	9.398	0.000	3.254	9.662	10.800
**Diagnostics**							
R2	0.873	Lambda	0.155	Sigma	3	Looloss	10.459

**Note:** * stands for the significance at the 1% level.

**Fig 8:**
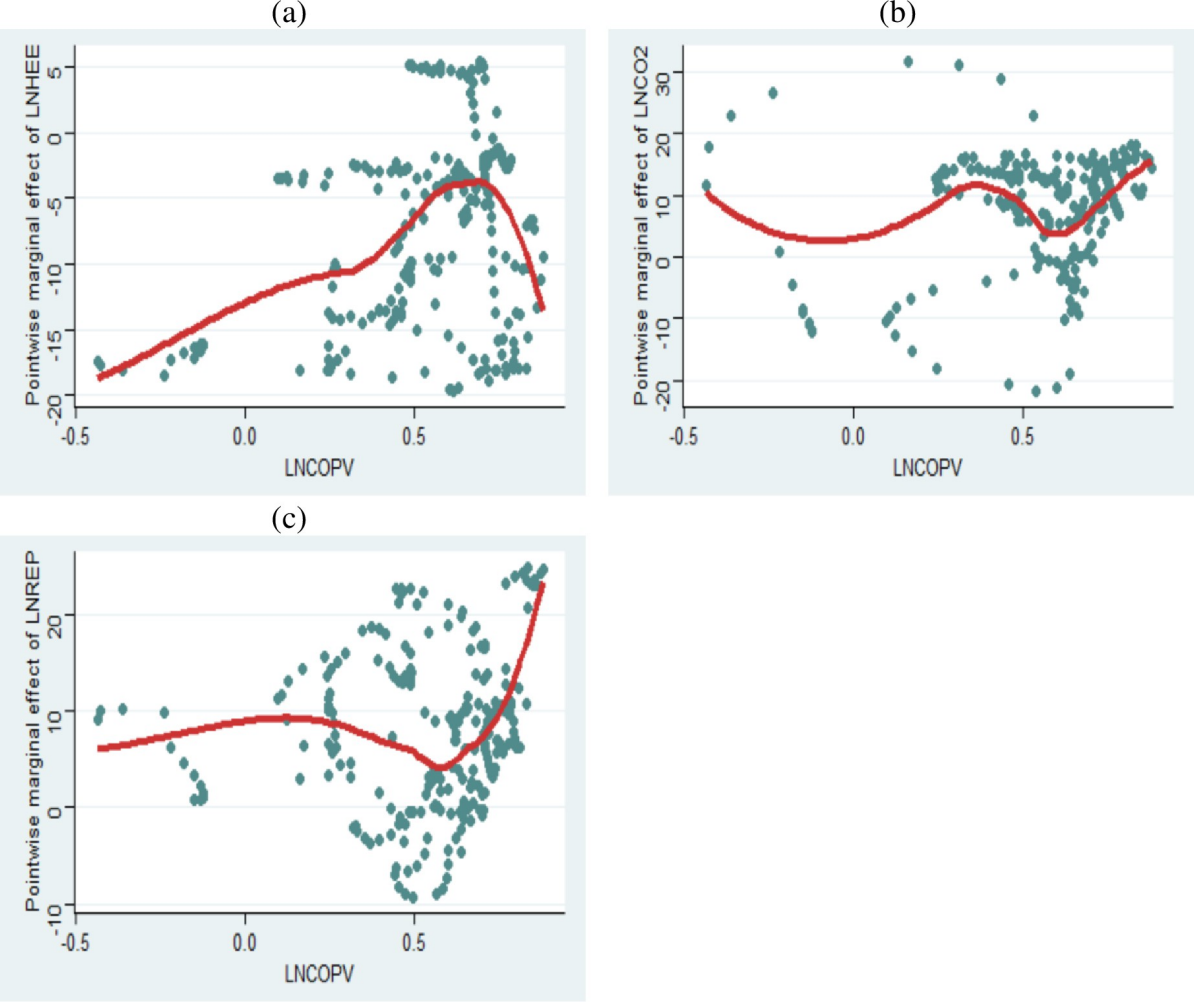
Marginal effect plots with flexible KLRS machine learning approach. Panel (a) is the Marginal effect of LNHEE on LNCOPV. Panel (b) is the Marginal effect of LNCO2 influences on LNCOPV. Panel (c) is the Marginal effect of LNREP on LNCOPV. The horizontal axes of the panels depict the quantiles of LNCOPV.

In addition, at each data point of LNCOPV, we estimate the marginal effect of LNHEE, LNCO_2_, and LNREP as shown in the marginal effect plots obtained via a flexible KLRS machine learning approach. The results as shown in Fig 8 confirm the robustness of the findings based on the multivariate-QQR models. From Fig 8(A), we find that the marginal effect of the LNHEE on the data point of LNCOPV is positive. This confirms that LNCOPV responds negatively to changes in LNHEE. Conversely, Fig 8(B) and 8(C) suggest that the marginal effects of LNCO_2_ and LNREP are positive. This perhaps confirms that LNCOPV responds positively to changes in LNCO_2_ and LNREP as already revealed by our results.

### 5. Conclusion and policy implications

Crude oil price volatility has become a distinguishing characteristic of the world’s economy since the advent of the energy crisis in 1970s due to turmoil in the Middle East. This has been exacerbated by the recent pandemic caused by COVID-19 which exerts pressure on the international oil market to experience unstable oil prices in a way that hurts the economies of the world like never before. Even though several studies have attempted to test the effect of oil price shocks on macroeconomics, there is still a paucity of literature, particularly on how crude oil price volatility responds to improvement in household energy use. To undertake this task, we apply the multivariate-QQR model using not only the historical dataset but also the forecast dataset, covering the period 1970Q1–2040Q4. Following the results obtained for this study, it is clear that the improvement in LNHEE dampens LNCOPV, and the effect is stronger in quantiles before the median quantiles. These outcomes further suggest that the influence of LNHEE decreases with increasing quantiles of the LNCOPV. Furthermore, the effect of both LNCO_2_ and LNREP intensify LNCOPV with evidence of asymmetric effect across quantiles. For example, while LNCO_2_ is stronger in the upper tails of the lower and higher quantiles, the LNREP is stronger in the higher quantiles of LNCOPV.

Therefore, from a policy perspective, the following recommendations are suggested to circumvent crude oil price volatility via the channel of an improvement in household energy use: First, since household energy efficiency dampens volatility in crude oil prices, we suggest the need to foster energy efficiency campaigns by the government administrators in the United States in order to pursue strategies and initiatives that prioritize energy efficiency programs at the level of household. This initiative can be incentivized through tax rebates or subsidies for energy-efficient appliances. In addition to fostering energy efficiency campaigns, we recommend the need for the government administrators to promote energy-efficient practices by strengthening existing energy efficiency codes and standards to guarantee effective regulation and ensuring that a minimum standard for efficient use of energy is retained. By implication, this will ensure cost-effectiveness in energy utilization and environmental protection as well as dampen over-reliance on crude oil reduce crude oil price volatility.

Furthermore, given the positive effect of energy-related CO_2_ emissions and retail-electricity prices on crude oil price volatility, there is a need for investments in renewable energy options. In other words, governments and policymakers need to prioritize investment in renewables such as photovoltaic, solar energy, wind energy, and hydro energy sources which are well-known cleaner energy resources that are more environmentally friendly relative to their counterparts (fossil fuel-based energy resources). This calls for the need for a paradigm shift in energy portfolio diversification in the United States’ energy mix. Shifting towards alternative energy is a pathway to mitigate the adverse effects of conventional energy consumption and promote environmental sustainability in the United States. This proposition aligns with the United States energy and sustainability targets for cleaner ecosystems, which also coincides with the United Nations Sustainable Development Goals that highlight the need for access to clean and sustainable energy (UNSDG-7) and climate change action (UNSDG-13).

Additionally, from policy optics, there is a need to reinforce international collaborations in line with the UNSDG-17, which advocates global partnerships for goals. This suggestion is relevant given the nature of the crude oil market in managing price variances. There is a need for countries to collaborate on the following fronts namely technological transfer, global best practices, and joint research and development agenda to advance energy efficiency and promote strategies that mitigate over-reliance on crude oil. Furthermore, there is a need to implement carbon taxing mechanisms such as certain tax systems for polluters at a certain threshold to curb the effect of CO_2_ emissions from conventional energy.

Although the present study explores the reaction of crude oil price volatility to household energy-related efficiency and assesses the pathways to sustainable crude oil prices and energy-efficient practices in the United States, there might be a need for future studies to explore how energy-related efficiency in major oil exporters like the Kingdom of Saudi Arabia (KSA) helps mitigate volatility in crude oil prices. This will provide a comprehensive understanding of this subject matter, which is important for achieving environmental sustainability.

## Supporting information

S1 File Study highlight. (DOCX)

S1 Data Data file. (XLSX)
