# Supplementary material for: Can household energy efficiency dampen crude oil price volatility in the United States?
Source: PLoS One. 2025 Jan 21;20(1):e0307840. doi: 10.1371/journal.pone.0307840 (PMC11750086; doi:10.1371/journal.pone.0307840)
Supplement: S1 File — (DOCX) [file pone.0307840.s001.docx]

**Highlights**

- Household energy efficiency (LNHEE) and crude oil price volatility (LNCOPV) in the U.S.
- Nonparametric multivariate quantile-on-quantile regression model is applied
- LNHEE lowers LNCOPV with stronger effect in quantiles before the median quantiles of LNCOPV
- Energy-related CO_2_ emissions and retail electricity prices asymmetrically dampen LNCOPV across quantiles.
- Negative effect of LNHEE decreases with increasing quantiles of the LNCOPV
